# EEG complexity correlates with residual consciousness level of disorders of consciousness

**DOI:** 10.1186/s12883-023-03167-w

**Published:** 2023-04-03

**Authors:** Yangfeng Liu, Wentao Zeng, Na Pan, Xiaoyu Xia, Yonghua Huang, Jianghong He

**Affiliations:** 1 Xijing 986 Hospital Department, Fourth Military Medical University, Xi’an, China; 2 The Seventh Medical Center of PLA General Hospital, Beijing, China; 3 Department of Medical Imaging, The First Affiliated Hospital of Xi’an Jiaotong University, Xi’an, China; 4 Department of Neurosurgery, Beijing Tiantan Hospital, Capital Medical University, Beijing, China

**Keywords:** Lempel–Ziv complexity, EEG, Disorders of consciousness, Minimally conscious state

## Abstract

**Background and objective:**

Electroencephalography (EEG) and neuroimaging measurements have been highly encouraged to be applied in clinics of disorders of consciousness (DOC) to improve consciousness detection. We tested the relationships between neural complexity measured on EEG and residual consciousness levels in DOC patients.

**Methods:**

Resting-state EEG was recorded from twenty-five patients with DOC. Lempel–Ziv complexity (LZC) and permutation Lempel–Ziv complexity (PLZC) were measured on the EEG, and their relationships were analyzed with the consciousness levels of the patients.

**Results:**

PLZC and LZC values significantly distinguished patients with a minimally conscious state (MCS), vegetative state/unresponsive wakefulness syndrome (VS/UWS), and healthy controls. PLZC was significantly correlated with the Coma Recovery Scale-Revised (CRS-R) scores of DOC patients in the global brain, particularly in electrodes locating in the anterior and posterior brain regions. Patients with higher CRS-R scores showed higher PLZC values. The significant difference in PLZC values between MCS and VS/UWS was mainly located in the bilateral frontal and right hemisphere regions.

**Conclusion:**

Neural complexity measured on EEG correlates with residual consciousness levels of DOC patients. PLZC showed higher sensitivity than LZC in the classification of consciousness levels.

**Supplementary Information:**

The online version contains supplementary material available at 10.1186/s12883-023-03167-w.

## Introduction

Disorders of consciousness (DOC) are highly heterogeneous clinical entities. After severe brain injury, some patients with coma turn to a vegetative state (VS) [1], also known as unresponsiveness wakefulness syndrome (UWS) [2], which is characterized by spontaneous eye opening despite no self or environmental awareness. UWS patients may remain in that state permanently, evolve into a minimally conscious state (MCS) (i.e., minimal but definite behavioral evidence of self or environmental awareness), or emerge from a minimally conscious state (EMCS) (i.e., functional communication or object use) [3]. The level of consciousness has clinical and ethical implications for the outcome prediction, rehabilitation scheme, resource allocation, and end-of-life decisions of DOC patients [4–6]. Standardized behavioral assessments are the most common methods because they are availability and intuitive correlation of consciousness [7, 8]. The results of these assessments are often misled by injured motor function, fluctuated arousal, or other comorbidities [9].

Other than behavioral assessments, neuroimaging techniques are also indicative of residual consciousness in DOC patients and are free of motor function or active participation (when assessed in a resting state); thus, they can be important supplements to behavioral assessments [10–13]. Evidence and theoretical models suggest that synergy neural activities between regions with enough complexity are needed to maintain consciousness [11, 13–15]. Lempel–Ziv complexity (LZC), a classic complexity metric, is widely used in a variety of applications [16]. LZC reduction in the electroencephalogram (EEG) was observed in subjects with different types of consciousness loss (e.g., anesthesia, epileptic seizure, and DOC) [17–19]. Recent studies have even found that LZC is significantly correlated with the distribution of the power density spectrum of coma patients and patients under anesthesia [20, 21].

However, LZC is easily affected by noise and loses much information for a fairly rough coarse-graining process. Bai et al. [19] invented a new index by improving the coarse-graining process of LZC based on a permutation method (the permutation Lempel–Ziv complexity [PLZC]). The performance of LZC and PLZC in differentiating consciousness levels against strong noise was tested in simulated EEG signals mixed with white noise. PLZC showed better performance than LZC, even in a high-noise setting [19]. Despite these simulation results, a significant correlation between PLZC and drug effects has been reported in patients under anesthesia [19, 22]. These findings encourage the assumption that PLZC may be a good indicator of neural complexity and can be an effective consciousness biomarker.

Therefore, the aim of this study is to investigate the correlation of PLZC and consciousness levels of DOC patients. We hypothesize that PLZC would perform better than LZC in detecting the residual consciousness levels of DOC patients.

## Methods

### Patients and healthy controls

DOC patients were enrolled into the study from the Seventh Medical Center of the Chinese PLA General Hospital based on the inclusion criteria: (i) persistence of a DOC state for at least 1 month after the acute brain insult; (ii) stable vital signs and free of acute medical complications (e.g., acute pneumonia). Exclusion criteria were conditions unfit for EEG acquisition (e.g. craniotomy and affects EEG signal), unstable clinical conditions, and the effect of treatments or drugs on cortical excitability during CRS-R assessments or EEG acquisition. Twenty-five patients (9 females, mean age 50 ± 13.1 years, mean time since injury 4.3 ± 2.3 months, 8 with traumatic etiology, 17 with stroke etiology) and healthy controls were recruited for this study. The demographic and behavioral data of the patients are reported in Table 1. Inclusion criteria were DOC diagnosed with the Coma Recovery Scale-Revised (CRS-R) [23] and an age above 18 years old. The study was approved by the ethics committee of the Seventh Medical Center of the Chinese PLA General Hospital. Informed consent to participate in the study was obtained from the legal surrogates of the DOC patients and healthy controls (Additional file 1. Figure S1).

**Table 1:** Demography of the patients

Patient	Age	Gender	Etiology	Time since injury (months)	CRS-R	Diagnosis
1	26	F	Stroke	4	6	**VS/UWS**
2	60	M	TBI	8	6	**VS/UWS**
3	39	M	Stroke	3	17	**MCS**
4	42	M	Stroke	2	7	**MCS**
5	44	M	Stroke	1.5	10	**MCS**
6	43	M	TBI	8	4	**VS/UWS**
7	46	M	Stroke	2.5	5	**VS/UWS**
8	57	F	Stroke	2	4	**VS/UWS**
9	23	M	TBI	2	7	**MCS**
10	47	M	Stroke	2	6	**VS/UWS**
11	67	M	Stroke	2	5	**VS/UWS**
12	52	M	Stroke	4	5	**VS/UWS**
13	47	F	Stroke	6	8	**MCS**
14	53	M	Stroke	8	11	**MCS**
15	68	M	TBI	1.5	11	**MCS**
16	49	F	TBI	1.5	12	**MCS**
17	54	M	Stroke	8	8	**MCS**
18	33	M	Stroke	4	5	**VS/UWS**
19	53	M	TBI	7	10	**MCS**
20	36	M	TBI	5	10	**MCS**
21	79	F	Stroke	6	7	**VS/UWS**
22	63	F	Stroke	5	8	**MCS**
23	64	F	Stroke	6	9	**MCS**
24	52	F	TBI	3	8	**MCS**
25	53	F	Stroke	5	3	**VS/UWS**

*M* Male, *F* Female, *TBI* Traumatic brain injury, *CRS-R* Coma recovery scale-revised, *VS/UWS* Vegetative state/unresponsive wakefulness syndrome, *MCS* Minimally conscious state

### CRS-R assessment

CRS-R was conducted once each day for each patient. Clinical diagnosis was established according to the best performance of repeated CRS-R (5 times) during 1 week, as suggested by Wannez et al. [24]. It is a standardized and validated scale that assesses the residual level of consciousness of severely brain-injured patients. The CRS-R consists of six subscales (auditory, motor, visual, oral-motor/verbal, communication, and arousal), each comprising items of increasing complexity that allow for the detection of subtle signs of consciousness (MCS) or functional communication or object use (EMCS).

### EEG acquisition and preprocessing

EEG data were continuously acquired from 62 scalp channels (Ag/AgCl-pin electrodes) arranged in the standard international 10–10 system (BrainAmp 64 MRplus. Brain Products, Munich, Germany). A band-pass filter at DC to 1 kHz was set at the recorder with a sampling rate of 1 kHz. The subjects were kept awake (eyes open) during EEG acquisition. The CRS-R arousal facilitation protocol was applied when signs of sleepiness (prolonged eye closure) were observed. Data recording was paused until signals recovery to baseline during arousal facilitation. The skin-to-electrode impedances were kept under 5 KΩ, while the sampling rate was set at 1 kHz.

Preprocessing of EEG data was performed offline with the EEGLAB 12.0.2.5b toolbox in MATLAB (Version R2014a, MathWorks Inc., Natick, USA) that included five steps: (1) identify and remove abnormal EEG segments caused by involuntary movements (e.g., coughing and biting); (2) filter EEG data to a bandwidth between 1 and 45 Hz with line noise removed by a 50 Hz notch filter; (3) downsample EEG data to 500 Hz; (4) use the independent component analysis (ICA) function to remove eye movement and muscle activation artifacts; (5) cut the artifact-free EEG data into 10 s epochs with a 50% overlap; and (6) select 25 epochs with visual inspection by an EEG expert and administer an average reference on them.

### Permutation Lempel–Ziv complexity

**Fig. 1:**
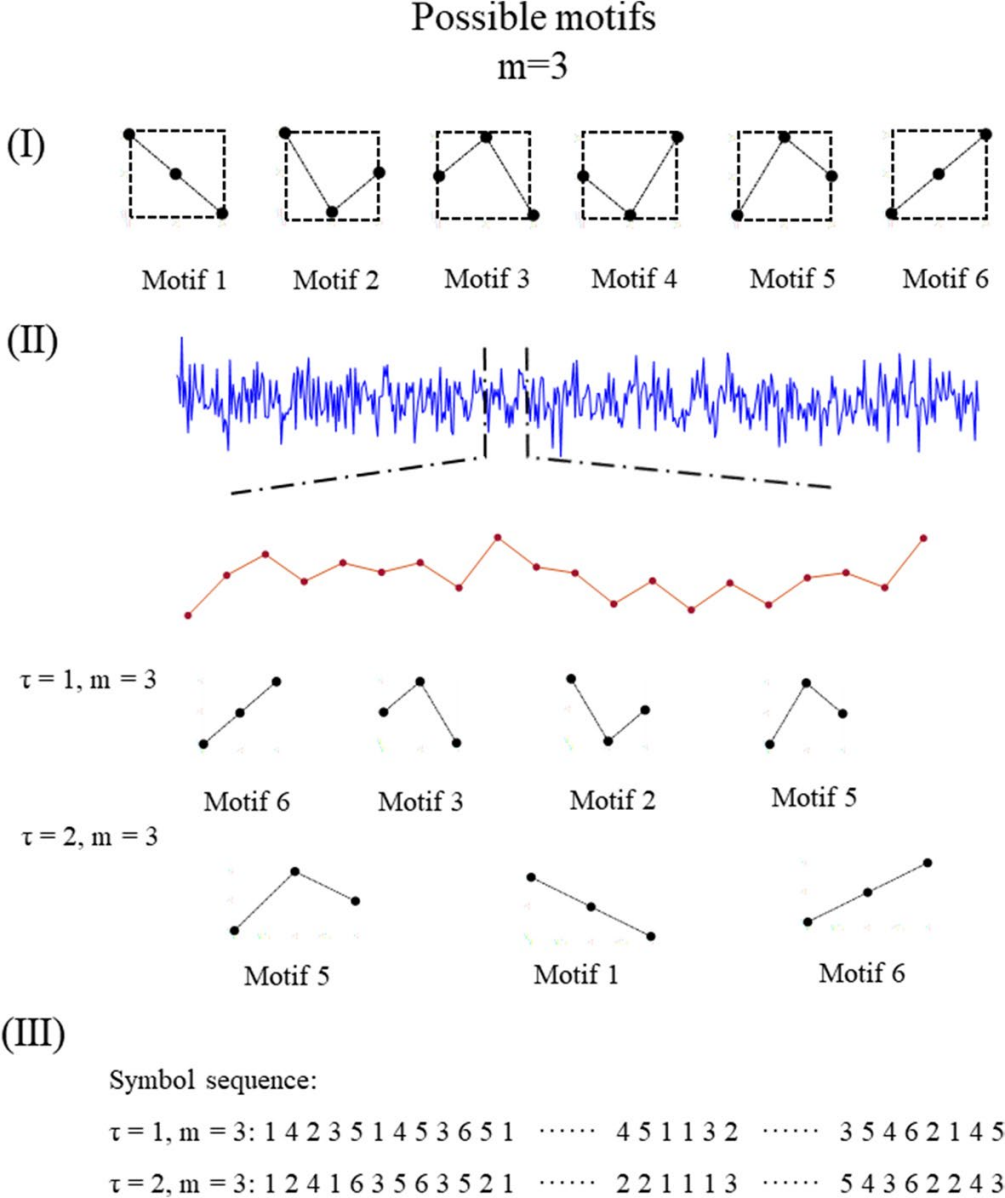
Permutation processing of transform from signal to symbolic sequence. (I) Possible motifs when m = 3 (II). A signal is segmented into motifs (III). The symbol sequence of the signal in (II)

The coarse-graining procedure in PLZC is based on permutation. Order patterns were used to transform the original EEG signal into a symbol sequence. The permutation scheme is illustrated in Fig. 1. Each EEG epoch was broken into segments from left to right with length m and interval τ for subsequent permutation. For example, in a sequence {S(n)} = S_1,_ S_2,_ S_3,_ S_4,_ S_5,_ S_6,_ S_7_ with S_n_ as the sample points, the segments are S_1_S_3_S_5_, S_2_S_4_S_6_, and S_3_S_5_S_7_ when m = 3 and τ = 2. The order pattern of each segment was determined by permutating the sample points according to their values. In the case of equal values, the latter sample point was considered as higher amplitude than the earlier. Each pattern was referred to as a motif and indexed with an integer. The length m thus determined the number of possible motifs as the factorial of m. It is easy to see that different m and τ make different segments and patterns. In this study, we chose m = 3 and τ = 1, following the criteria suggested by Bai et al. [19]. After the segmentation and permutation procedure, broadband EEG epochs were transformed into symbol sequences of motif indices.

Then, the complexity was calculated based on the Lempel–Ziv complexity with the primary parsing scheme (e.g., exhaustive history of a sequence) [16].

Step 1: Transform the original signal into a finite symbol sequence {x(n)} consisting of motif indices. No more than m ! symbols/motif indices would be presented in this sequence.

Step 2: Initialize the complexity index c(n) as 1, which indicates the number of innovative strings in the sequence. Generate S and Q as the first and second symbols, respectively.

Step 3: Concatenate S and Q to be a String SQ. Delete the last symbol of the SQ to be a new string SQv.

Step 4: Judge whether Q has reached the end of {x(n)}, and go to Step 8 if it has.

Step 5: Check if Q is a substring of SQv. Go to Step 6 in the positive case and Step 7 in the negative case. The former indicates that Q is not an innovative string.

Step 6: Keep S unchanged and concatenate the next symbol to Q as the new Q. Go to Step 3 to perform the comparison again.

Step 7: Increase c(n) by 1. Set SQ in Step 4 as new S and the next symbol as new Q. Go to Step 3 to compare the new SQv and Q.

Step 8: After the repeated comparison procedure, {x(n)} is now parsed into c(n) innovative strings. We then encoded all these innovative strings with their permutation indices. The length of the code sequence is thus calculated as $L(n)$:

1 $$L(n)=c(n)\lbrack log_{m!}c(n)+1\rbrack$$

The reason of $L(n)$ is that we need ${\mathrm{log}}_{m!}c\left(n\right)$ plus 1 bit to encode the prefix and the last symbol of each string when the size of alphabet is m! [25]. Then, PLZC output is defined as $L(n)$ normalized by $n$, the length of the symbol sequence:

2 $$PLZC =\frac{c(n)\lbrack log_m!\;c(n)+1\rbrack}n$$

When n is very large, $c(n)\le \frac{n}{{\mathrm{log}}_{m!}n}$ [16], (2) can thus be further simplified as follows:

3 $$PLZC=\frac{c(n)\;\lbrack log_m!\;n\rbrack}n$$

The procedure hereinbefore was performed in individual EEG epochs of patients and healthy controls.

### Lempel–Ziv complexity

The calculation of LZC is the same as PLZC except for the coarse-graining procedure. The coarse-graining procedure of the LZC is dichotomous and based on a threshold value. Data points with values higher than or equal to the threshold value are considered symbol 1, and others are considered symbol 0. In this study, we chose the median of each epoch as the threshold.

### Statistics

All statistical analyses were conducted using the Statistics Toolbox in MATLAB (Version R2014a, MathWorks Inc., Natick, USA). The global average LZC and PLZC were computed with each trial and averaged across all trials over all EEG electrodes. The significance of global LZC and PLZC between VS/UWS, MCS, and healthy controls was tested using the Mann–Whitney U test with Bonferroni correction for multiple comparisons. Pearson’s linear correlation was applied for the correlation analysis between the global average LZC and PLZC and the CRS-R scores of the DOC patients.

The PLZC/LZC values of the electrodes in each brain region were averaged to represent local complexity. Pearson’s linear correlation was applied for the correlation analysis between the local average LZC and PLZC and the CRS-R scores of the DOC patients. Then, differences in LZC and PLZC between group pairs were compared using the Mann–Whitney U test at the electrode level. The false discovery rate (FDR) was used for multiple comparison corrections. Furthermore, we performed a stepwise logistic regression analysis based on generalized linear model using binomial distribution model (*p* value > 0.05 was used as exclude criteria) and a Wald statistic to investigate the strongest classifier between MCS vs. VS/UWS among all the properties, including PLZC and LZC in five brain regions. To test the hypothesis that the PLZC classify DOC independent on individual characteristics, we additionally performed a Bayesian analysis of covariance (ANCOVA) as implemented in Jasp v. 0.11.1 with the patients group (MCS and VS/UWS) as dependent variable, PLZC as a fixed-factor, subject as a random factor, and the patients' etiology and time since brain injury as a covariate.

## Results

**Fig. 2:**
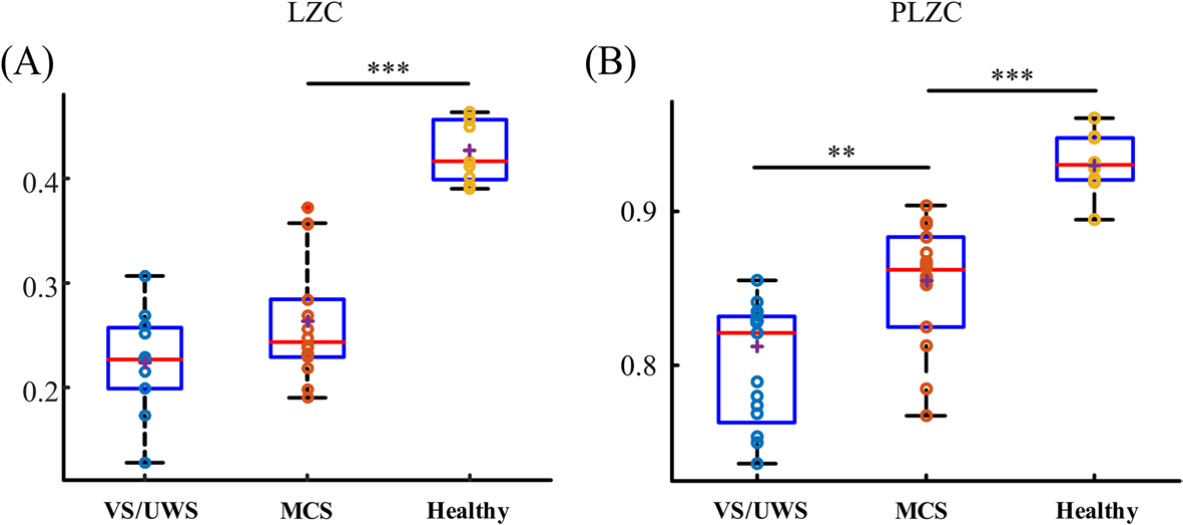
Boxplots of global average values of PLZC/LZC between minimally conscious state (MCS), vegetative state/unresponsive wakefulness syndrome (VS/UWS) and healthy control. *** indicates significant difference with *p* < 0.001; ** indicates significant difference with *p* < 0.01 after Bonferroni correction

Global LZC in healthy controls was significantly higher than in MCS (0.395 ± 0.037 vs. 0.269 ± 0.064, *p* < 0.001, U = 117), and no significant difference was found between MCS and VS/UWS (0.269 ± 0.064 vs. 0.212 ± 0.042, *p* = 0.355). The difference in global PLZC was significant both in healthy controls vs. MCS (0.913 ± 0.027 vs. 0.857 ± 0.036, *p* < 0.001, U = 115) and MCS vs. VS/UWS (0.857 ± 0.036 vs. 0.806 ± 0.039, *p* = 0.001, U = 96) (see Fig. 2) (Additional file 1. Table S1).

**Fig. 3:**
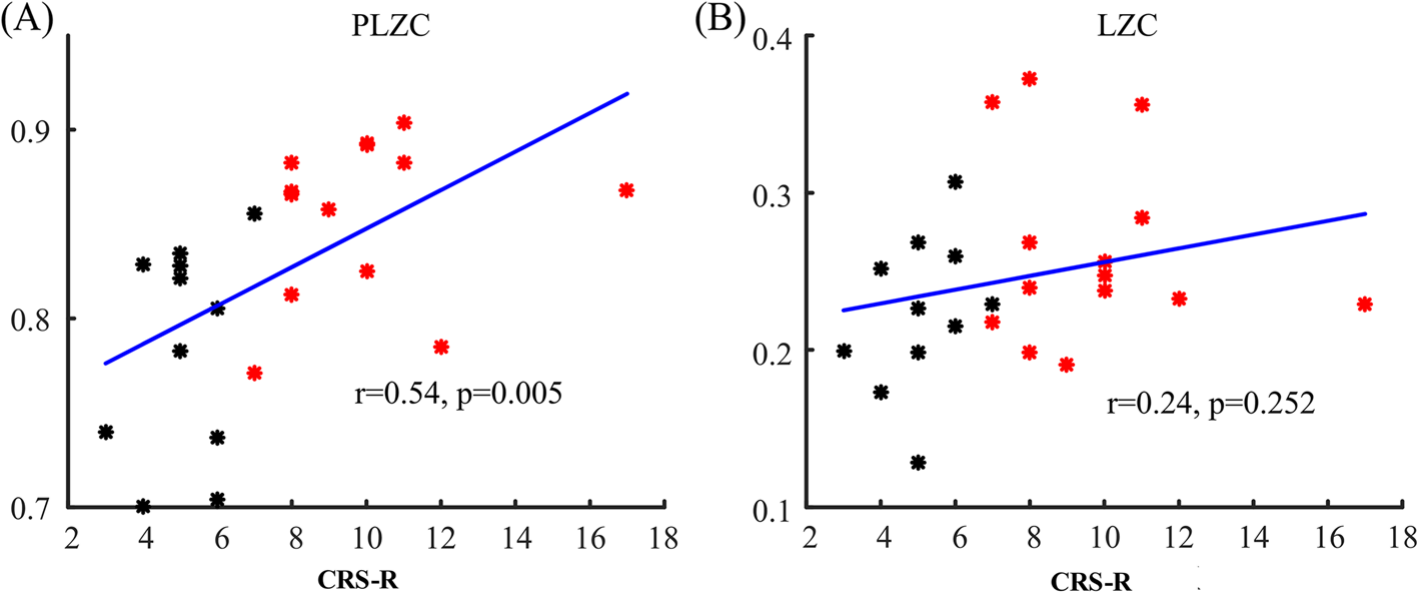
Pearson correlation of global average PLZC (**A**) and LZC (**B**) with CRS-R. Black shows patients with vegetative state/unresponsive wakefulness syndrome (VS/UWS) and red shows patients with minimally conscious state (MCS)

**Fig. 4:**
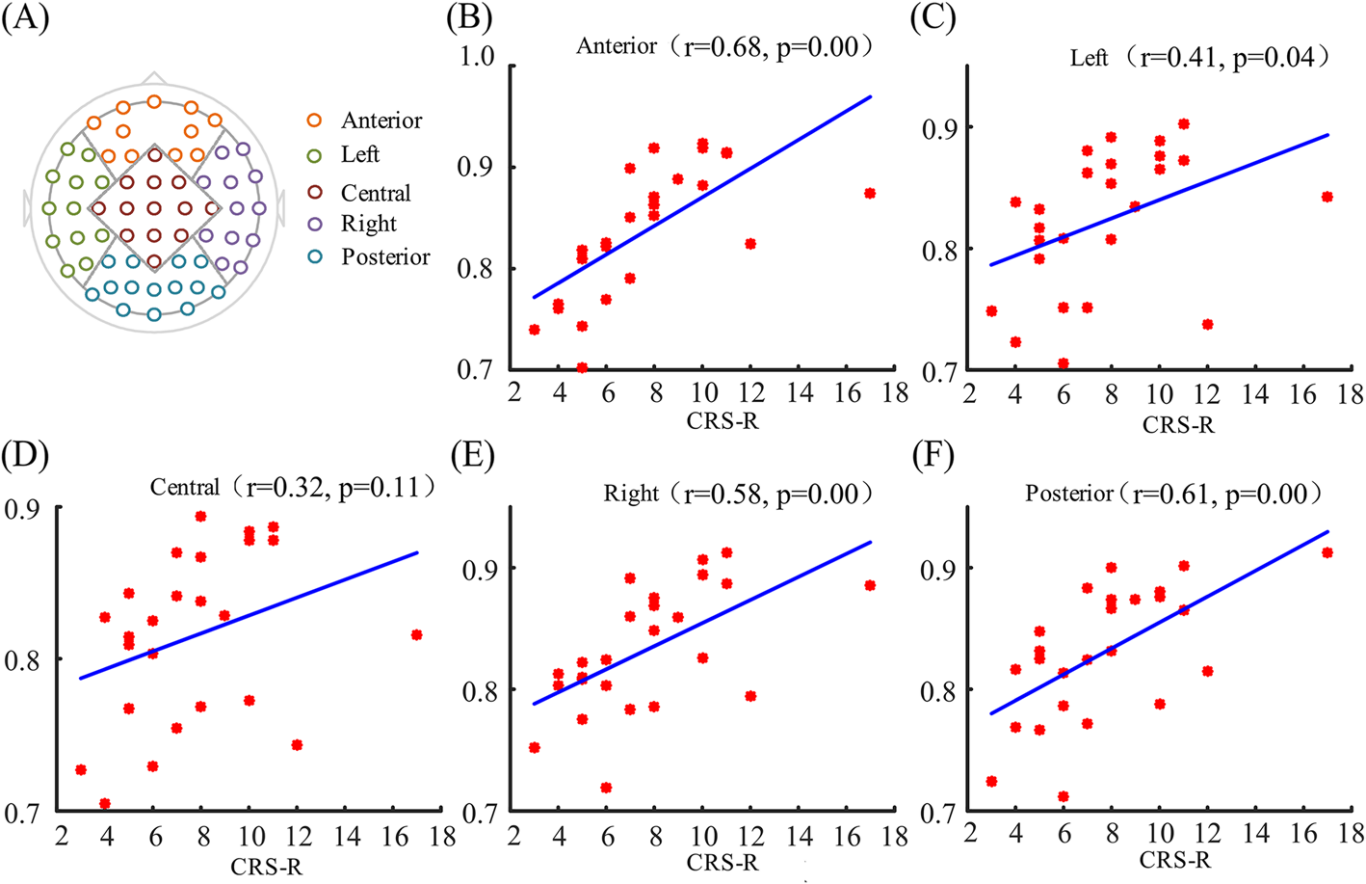
Pearson correlation of PLZC in brain regions and CRS-R. **A** Channels of the five identified brain regions. **B–F** Correlation of average PLZC in each brain region with CRS-R

The global average PLZC values showed a significant correlation (*r* = 0.54, *p* = 0.005) with the CRS-R scores. LZC showed a positive correlation trend but without significance (*r* = 0.24, *p* = 0.252) with CRS-R scores (see Fig. 3). Five brain regions of interest were defined based on EEG electrodes: anterior, central, left, right, and posterior (Fig. 4A). The average PLZC values in all defined brain regions except central showed a significant correlation with CRS-R values (Fig. 4). Among them, PLZC in the anterior (*r* = 0.68, *p* < 0.001) and posterior (*r* = 0.61, *p* < 0.001) regions showed highly significant correlations with CRS-R scores. Moreover, right brain regions (*r* = 0.58, *p* < 0.001) also showed highly significant correlations with CRS-R scores as compared to the left side (*r* = 0.41, *p* = 0.04).

**Fig. 5:**
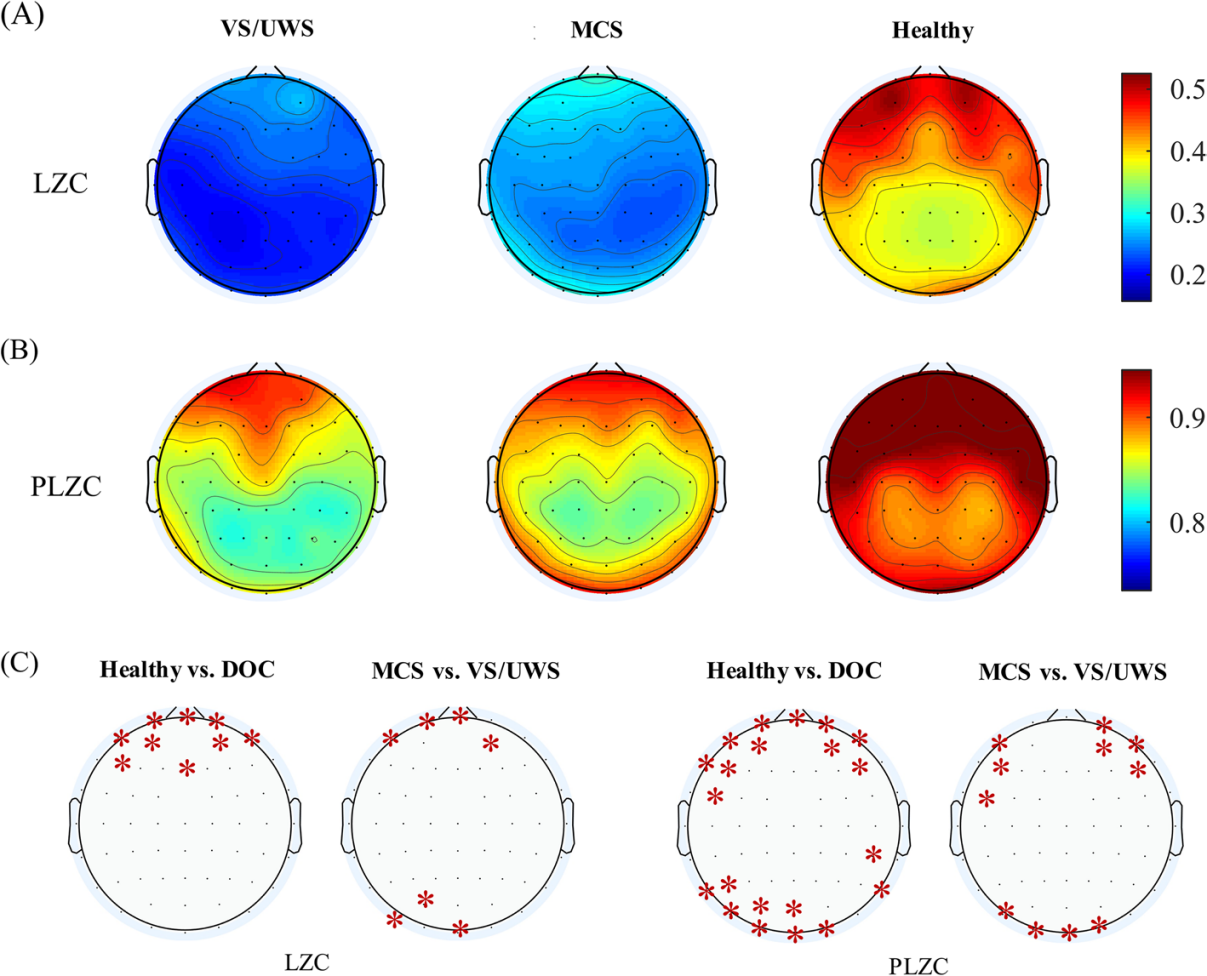
Topoplots of group average values of PLZC (**A**) and LZC (**B**) in minimally conscious state (MCS), vegetative state/unresponsive wakefulness syndrome (VS/UWS) and healthy control. The colorbar shows the PLZC and LZC values; dark red indicates a high value, and dark blue indicates a low value. **C** Electrodes (red *) with significantly different LZC/PLZC values between group pairs after FDR correction

Figure 5 shows the group average topoplot of LZC and PLZC at the electrode levels of MCS, VS/UWS, and healthy controls. Healthy controls showed distinctly higher PLZC and LZC values overall than DOC patients. The LZC and PLZC values in the anterior and posterior regions of MCS were higher than VS/UWS. Statistical analysis showed that significant differences in LZC and PLZC values between healthy vs. DOC and MCS vs. VS/UWS were primarily located in the anterior and posterior regions. PLZC highlighted more significantly different electrodes in the pairwise comparison group than did LZC.

A stepwise logistic regression analysis indicated that the strongest classifier (Chi2stat = 20.80, *p* < 0.001) between MCS vs. UWS/VS was PLZC of anterior brain region. Wald statistic confirmed that only PLZC of right hemisphere (Wald statistic = 9.28, *p* = 0.002) but not any LZC, could potentially contribute to the classification model. Bayesian ANCOVA excluded relevant probabilities (all BF < 1) of using etiology or time since brain injury as covariate of anterior PLZC MCS vs. VS/UWS classification. No individual clinical difference showed evidence (BF > 3) of covariate in the MCS vs. VS/UWS classification using PLZC.

## Discussion

We showed that the LZC and PLZC values were significantly different between groups with different consciousness levels. The LZC-based complexity algorithm describes the complexity (contents of information) of neural activities by measuring the rate of appearance of new patterns in the EEG series. Neural complexity is one of the necessary bases of consciousness, according to the integrated information theory proposed by Tononi [14]. Our result consistently supports integrated information theory and further suggests that PLZC will perform better than LZC in correlation with the residual consciousness of DOC patients. The global LZC was observed in a recent study with LZC variation between VS/UWS and MCS presented only at the theta band [26]. The underlying reason for this may be explained by the rather rough coarse-graining procedure of the LZC. The spectral distribution of EEG represents the structure of rhythmic neural activities, which alternate significantly either in temporal or prolonged consciousness loss [20, 27]. LZC, despite its time domain nature, was correlated with spectral distribution in both healthy controls and patients with altered consciousness [20, 21]. However, the dichotomous coarse-graining procedure of the LZC binarizes the original EEG sequence according only to a threshold. By doing so, data points corresponding to higher frequency variations would be assigned the same symbol due to lower amplitudes, thus losing information aspects meaningful to the differential diagnosis of DOC. In contrast, the permutation method used in PLZC breaks the original signals into motifs, which indicates the mutual relations of data points. Although spends more computation cost, PLZC retains the variations in both high and low frequencies into motifs and helps extract the minimal difference between VS/UWS and MCS. The consciousness of MCS patients is characterized as minimal and often misdiagnosed as VS/UWS [3]. It is logical that not all EEG features that differentiate healthy controls and DOC patients successfully do the same in MCS and VS/UWS. Our findings indicate that PLZC has better potential than LZC in the differential diagnosis of DOC.

At the local level, PLZC, in all except the central region, significantly correlated with CRS-R values, with the highest r values presented in the frontier and posterior regions. The PLZC in central region also showed trend of positive correlation, however not significance because of individual outliers, with CRS-R values. PLZC at right hemisphere showed much closer relationship with CRS-R, as compared to left hemisphere. One of possible reason is that right-hemispheric brain regions predominantly modulate and interact with components of the ascending arousal system, which regulate behavioural arousal, consciousness and motivation, by receiving and processing information from it [28]. So, we consider that right-hemispheric PLZC might capture more consciousness relative difference of brain activities than left hemisphere. As evidenced by a large number of studies of neural correlates of consciousness (NCC), frontal areas are not directly responsible for consciousness experience but for related cognitive activities (e.g., attention allocation, task execution, and report) [29–32]. In contrast, a temporo-parietal-occipital hot zone (posterior hot zone) is closely associated with the minimal neural substrate essential for conscious perception [29, 30]. Our results showed a significant correlation between CRS-R scores and PLZC in both the frontal areas and the posterior hot zone. Considering the information nature of PLZC, this result is logical: PLZC reflects the amount of information in the whole brain or particular regions, thus reflecting specific aspects of consciousness processing (cognitive vs. sensory). Anterior PLZC reflected the amount of information in frontal areas, and the extent of preserved consciousness-related cognitive functions also correlated with CRS-R scores. Note that CRS-R values reflect not just consciousness levels but also related functions, such as attention, execution, and arousal. PLZC values showed distinct difference within different brain regions (Fig. 5B). Electrodes in anterior region had higher PLZC values than central and posterior region, no matter in healthy controls or DOC patients. Since sensitivity of PLZC to oscillatory deviations, the regional difference might be caused by spectral difference of brain. The topoplots also showed significantly different posterior and anterior sites of PLZC between the groups. Healthy controls and MCS patients showed higher PLZC in both posterior hot zones and frontier areas than DOC and VS/UWS, respectively. This is consistent with the fact that these two groups not only had higher consciousness levels but also higher attention and execution functions than their opponents. These results suggest that PLZC may be measured region-wise using anterior PLZC as the biomarker of function and PLZC in the posterior hot zone as a consciousness marker. However, the poor spatial resolution of EEG restricted our assumptions about the underlying meaning of PLZC. Future studies using simultaneous EEG–fMRI recordings may be suitable to further investigate the nature of local PLZC. As PLZC is a resting measure independent of report and task participation, it is suitable for use in NCC studies to dissociate consciousness and related cognitive functions. However, the reported results were based on the EEG trials selected by visual inspection of an EEG expert. Automatic trial selection is always suggested if considering to transfer PLZC into clinic practice.

## Conclusion

This study showed that PLZC is globally correlated with the consciousness of DOC patients, with better performance than LZC in capturing the neural difference between VS/UWS and MCS at the group level. Findings at the local level consistent with consciousness hubs also supported our hypothesis. All these results indicate that PLZC has the potential to be a robust consciousness biomarker and functional recovery. Future studies with a large sample size should be conducted to test this conclusion.

## Supplementary Information

**Additional file 1.**

## Data Availability

The datasets generated and analysed during the current study are not publicly available due information security issues of individual health data but data are available from the corresponding author on reasonable request.

## Abbreviations

DOC: Disorders of consciousness
UWS: Unresponsiveness wakefulness syndrome
VS: Vegetative state
MCS: Minimally conscious state
EEG: Electroencephalography
CRS-R: The Coma Recovery Scale-Revised scores
LZC: Lempel–Ziv complexity
PLZC: Permutation Lempel–Ziv complexity
