# Supplementary material for: EEG complexity correlates with residual consciousness level of disorders of consciousness
Source: BMC Neurol. 2023 Apr 3;23:140. doi: 10.1186/s12883-023-03167-w (PMC10069047; doi:10.1186/s12883-023-03167-w)
Supplement: Supplementary file 1 — Additional file 1. [file 12883_2023_3167_MOESM1_ESM.docx]

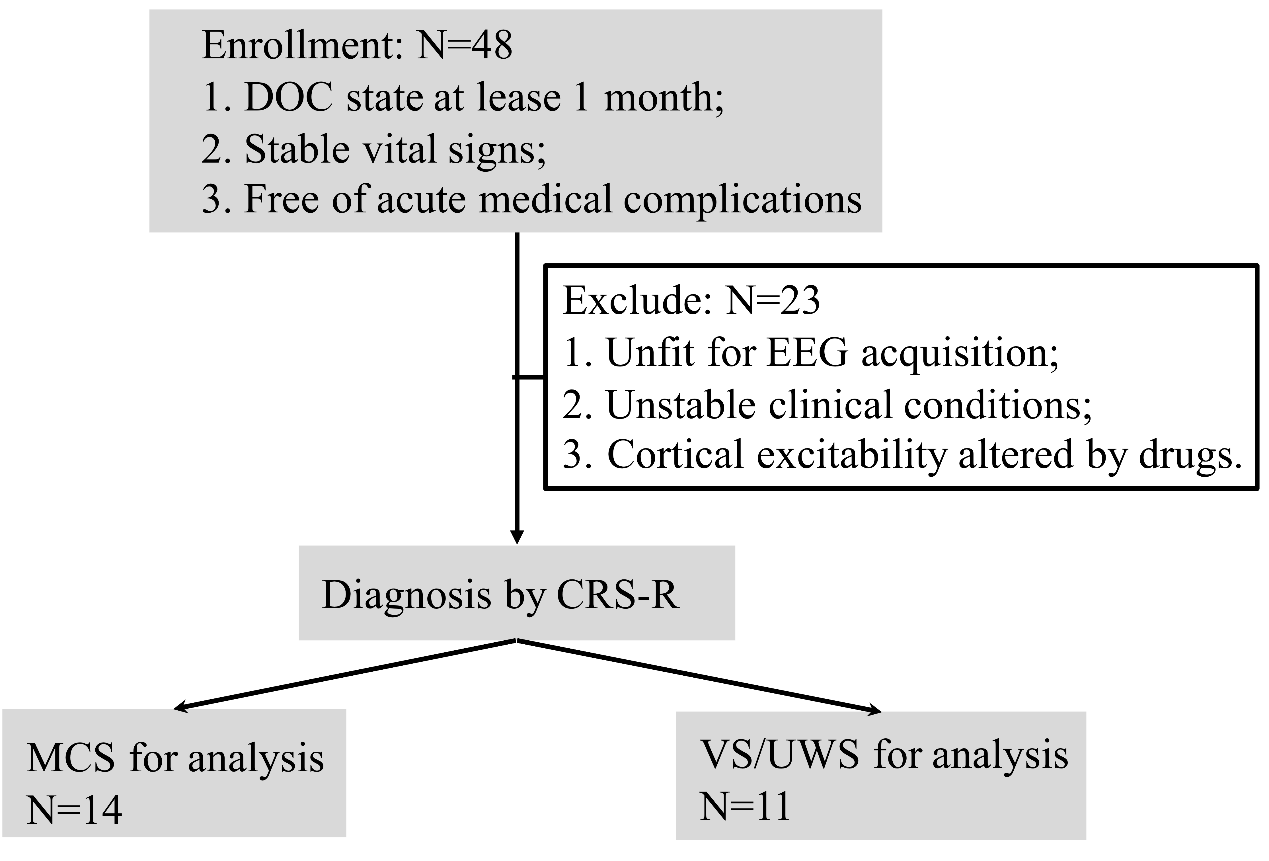


Figure S1. Study flow chart of the recruitment process. DOC: disorders of consciousness; VS/UWS: vegetative state/unresponsive wakefulness syndrome; MCS: minimally conscious state; CRS-R: Coma Recovery Scale-Revised scores.

Table S1. PLZC and LZC values of the patients in divided brain regions

| Patient  Number | Diagnosis | PLZC | | | | | LZC | | | | |
| --- | --- | --- | --- | --- | --- | --- | --- | --- | --- | --- | --- |
|  |  | A | L | C | R | P | A | L | C | R | P |
| 1 | **VS/UWS** | 0.825 | 0.808 | 0.803 | 0.803 | 0.786 | 0.287 | 0.186 | 0.242 | 0.311 | 0.252 |
| 2 | **VS/UWS** | 0.822 | 0.706 | 0.729 | 0.72 | 0.712 | 0.244 | 0.212 | 0.216 | 0.201 | 0.207 |
| 6 | **VS/UWS** | 0.761 | 0.723 | 0.705 | 0.813 | 0.816 | 0.196 | 0.168 | 0.186 | 0.159 | 0.159 |
| 7 | **VS/UWS** | 0.702 | 0.832 | 0.814 | 0.822 | 0.848 | 0.193 | 0.183 | 0.197 | 0.198 | 0.217 |
| 8 | **VS/UWS** | 0.765 | 0.838 | 0.827 | 0.803 | 0.769 | 0.308 | 0.248 | 0.264 | 0.233 | 0.208 |
| 10 | **VS/UWS** | 0.770 | 0.751 | 0.825 | 0.825 | 0.813 | 0.377 | 0.34 | 0.286 | 0.274 | 0.269 |
| 11 | **VS/UWS** | 0.810 | 0.817 | 0.843 | 0.81 | 0.831 | 0.115 | 0.119 | 0.141 | 0.133 | 0.132 |
| 12 | **VS/UWS** | 0.743 | 0.807 | 0.809 | 0.808 | 0.825 | 0.26 | 0.21 | 0.221 | 0.212 | 0.232 |
| 18 | **VS/UWS** | 0.819 | 0.792 | 0.767 | 0.775 | 0.767 | 0.332 | 0.277 | 0.231 | 0.27 | 0.24 |
| 21 | **VS/UWS** | 0.791 | 0.751 | 0.755 | 0.784 | 0.772 | 0.219 | 0.205 | 0.221 | 0.249 | 0.243 |
| 25 | **VS/UWS** | 0.740 | 0.749 | 0.727 | 0.752 | 0.725 | 0.212 | 0.203 | 0.196 | 0.206 | 0.178 |
| 3 | **MCS** | 0.775 | 0.743 | 0.716 | 0.785 | 0.812 | 0.239 | 0.213 | 0.214 | 0.23 | 0.247 |
| 4 | **MCS** | 0.899 | 0.862 | 0.841 | 0.86 | 0.824 | 0.239 | 0.211 | 0.213 | 0.225 | 0.206 |
| 5 | **MCS** | 0.924 | 0.876 | 0.878 | 0.907 | 0.876 | 0.303 | 0.271 | 0.221 | 0.225 | 0.228 |
| 9 | **MCS** | 0.851 | 0.881 | 0.87 | 0.892 | 0.883 | 0.331 | 0.453 | 0.335 | 0.271 | 0.394 |
| 13 | **MCS** | 0.863 | 0.807 | 0.768 | 0.786 | 0.831 | 0.208 | 0.201 | 0.188 | 0.178 | 0.21 |
| 14 | **MCS** | 0.915 | 0.873 | 0.878 | 0.887 | 0.865 | 0.408 | 0.336 | 0.347 | 0.363 | 0.33 |
| 15 | **MCS** | 0.914 | 0.902 | 0.887 | 0.913 | 0.901 | 0.306 | 0.244 | 0.264 | 0.298 | 0.298 |
| 16 | **MCS** | 0.825 | 0.737 | 0.743 | 0.794 | 0.815 | 0.236 | 0.214 | 0.217 | 0.225 | 0.261 |
| 17 | **MCS** | 0.919 | 0.892 | 0.867 | 0.875 | 0.867 | 0.332 | 0.277 | 0.231 | 0.27 | 0.24 |
| 19 | **MCS** | 0.883 | 0.865 | 0.773 | 0.826 | 0.788 | 0.257 | 0.284 | 0.241 | 0.242 | 0.26 |
| 20 | **MCS** | 0.919 | 0.889 | 0.884 | 0.894 | 0.88 | 0.305 | 0.211 | 0.232 | 0.233 | 0.211 |
| 22 | **MCS** | 0.852 | 0.87 | 0.894 | 0.868 | 0.9 | 0.209 | 0.215 | 0.215 | 0.253 | 0.298 |
| 23 | **MCS** | 0.888 | 0.834 | 0.829 | 0.859 | 0.874 | 0.207 | 0.174 | 0.181 | 0.185 | 0.202 |
| 24 | **MCS** | 0.871 | 0.853 | 0.838 | 0.849 | 0.874 | 0.369 | 0.435 | 0.364 | 0.309 | 0.386 |

VS/UWS: vegetative state/unresponsive wakefulness syndrome; MCS: minimally conscious state; A: anterior; L: left hemisphere; C: central; R: right hemisphere; P: posterior;
